# Feasibility, Acceptability, and Preliminary Efficacy of a Positive Affect Skills Intervention for Adults With Fibromyalgia

**DOI:** 10.1093/geroni/igad070

**Published:** 2023-07-13

**Authors:** Anthony D Ong, Kenneth Tyler Wilcox, Judith T Moskowitz, Elaine Wethington, Elizabeth L Addington, Mubarak O Sanni, Patricia Kim, M Cary Reid

**Affiliations:** Department of Psychology, Cornell University, Ithaca, New York, USA; Division of Geriatrics and Palliative Medicine, Weill Cornell Medical College, New York City, New York, USA; Department of Psychology, Cornell University, Ithaca, New York, USA; Department of Medical Social Sciences, Northwestern Feinberg School of Medicine, Chicago, Illinois, USA; Department of Psychology, Cornell University, Ithaca, New York, USA; Division of Geriatrics and Palliative Medicine, Weill Cornell Medical College, New York City, New York, USA; Department of Medical Social Sciences, Northwestern Feinberg School of Medicine, Chicago, Illinois, USA; Division of Geriatrics and Palliative Medicine, Weill Cornell Medical College, New York City, New York, USA; Division of Geriatrics and Palliative Medicine, Weill Cornell Medical College, New York City, New York, USA; Division of Geriatrics and Palliative Medicine, Weill Cornell Medical College, New York City, New York, USA

**Keywords:** Chronic pain, Positive affect, Positive psychology

## Abstract

**Background and Objectives:**

To examine the feasibility, acceptability, and preliminary efficacy of a positive affect skills intervention for middle-aged and older adults with fibromyalgia syndrome (FMS).

**Research Design and Methods:**

Ninety-five participants with FMS aged 50 and older (94% female) were randomized to 1 of 2 conditions: (a) Lessons in Affect Regulation to Keep Stress and Pain UndeR control (LARKSPUR; *n* = 49) or (b) emotion reporting/control (*n* = 46). LARKSPUR included 5 weeks of skill training that targeted 8 skills to help foster positive affect, including (a) noticing positive events, (b) savoring positive events, (c) identifying personal strengths, (d) behavioral activation to set and work toward attainable goals, (e) mindfulness, (f) positive reappraisal, (g) gratitude, and (h) acts of kindness. Outcome data were collected via online surveys at baseline, postintervention, and 1-month follow-up.

**Results:**

Completion rates (88%) and satisfaction ratings (10-point scale) were high (LARKSPUR: *M* = 9.14, standard deviation (*SD*) = 1.49; control: *M* = 8.59, *SD* = 1.97). Improvements were greater in LARKSPUR participants compared with control participants on measures of positive affect (Cohen’s *d* = 0.19 [0.15, 0.24]), negative affect (Cohen’s *d* = −0.07 [−0.11, −0.02]), and pain catastrophizing (Cohen’s *d* = −0.14 [−0.23, −0.05]). Improvements in positive affect (Cohen’s *d* = 0.17 [0.13, 0.22]) and negative affect (Cohen’s *d* = −0.11 [−0.15, −0.06]) were maintained at 1-month follow-up. Dose–response analyses indicated that intervention engagement significantly predicted pre-to-post and post–to-follow-up reductions in pain catastrophizing.

**Discussion and Implications:**

The current preliminary findings add to existing literature and highlight the specific potential of internet-delivered positive affect skills programs for adults with FMS.

**Clinical Trial Registration:**

NCT04869345.

**Translational Significance:** This study demonstrates the feasibility, acceptability, and preliminary efficacy of an internet-based intervention program to support positive affect skills training among middle-aged and older adults living with fibromyalgia syndrome (FMS). Findings indicate that for individuals with FMS, augmenting positive affect may be crucial for optimizing the effects of interventions in real-world settings. These findings are of interest to primary health care providers who serve vulnerable older adult populations, whose physical impairment and chronic pain may make access to traditional face-to-face clinical care difficult.

## Background and Objectives

Fibromyalgia syndrome (FMS) is a chronic pain disorder characterized by widespread musculoskeletal pain, fatigue, impaired sleep, and cognitive dysfunction (Hauser & Wolfe, 2012; Wolfe et al., 2010). Depression and deficits in positive affective states are present in a majority of individuals affected by FMS (Finan et al., 2009; Hassett et al., 2008; van Middendorp et al., 2008; Zautra et al., 2005). Conventional pharmacological treatments are only modestly effective, with many affected individuals experiencing undesirable side effects (Calandre et al., 2015; Clauw, 2014; Mease, 2005). Standard behavioral therapies, such as cognitive-behavioral therapy for pain (CBT-P; Morley et al., 1999) and mindfulness-based stress reduction (Grossman et al., 2007), typically focus on reducing negative affective states (e.g., anxiety and depression; Keefe et al., 2001; Koechlin et al., 2018) and yield only modest treatment benefits (Eccleston et al., 2013; Garcia-Campayo et al., 2008). Efforts are therefore needed to develop more effective approaches for FMS by identifying new targets for intervention.

Increasing evidence suggests that positive affective states (e.g., gratitude and happiness) play a unique role in promoting adaptive functioning in the face of chronic pain (Finan & Garland, 2015; Hausmann et al., 2014; Muller et al., 2016; Ong et al., 2015; Peters et al., 2017). Positive affect (PA) has been theorized to have a range of proximal benefits, such as motivating adaptive coping behaviors (Folkman, 1997; Tugade et al., 2004), countering the effects of negative affect (NA; e.g., fear and anxiety; Hanssen et al., 2017; Vlaeyen et al., 2016), reducing emotional reactivity to daily stress (Ong et al., 2006, 2009), minimizing negative pain-related cognitions, such as pain catastrophizing (Furrer et al., 2019; Ong et al., 2010), and fostering flexible responses to situational challenges (Garland et al., 2010; Kalisch et al., 2015). A meta-analysis of 29 observational and experimental studies documented a protective effect of PA on pain severity in adults with chronic noncancer pain (Ong et al., 2020). These findings are in line with a recent meta-analysis of randomized controlled trials (RCTs) showing beneficial effects of positive psychology interventions (PPIs) in the management of chronic pain (Braunwalder et al., 2021).

Although evidence to date supports the use of PPIs in the treatment of chronic pain (Braunwalder et al., 2021; Iddon et al., 2016), implementation of PPI approaches into clinical practice has been slow and varied (Finan & Garland, 2015; Ong et al., 2015). Potential barriers include high patient burden, privacy concerns, time, and mobility or travel limitations (Ariza-Mateos et al., 2020; Muller et al., 2016). Internet-delivered interventions for chronic pain can potentially overcome some of the access barriers to traditional face-to-face care, while assisting health care providers in disseminating programs to a wider population (Bender et al., 2011). Scalable interventions that can be delivered virtually are especially pertinent given calls for more research on the role of internet-based interventions for use with individuals 65 years and older (Bender et al., 2011) and with specific pain conditions, such as FMS that can cause fatigue and limit mobility (Friesen et al., 2017; Lorig et al., 2008; Williams et al., 2010).

To date, RCTs of internet-delivered pain management programs have focused on individuals with a diverse range of pain-related disorders, with few interventions examining subgroups of individuals with specific pain conditions, such as FMS, and none targeting enhancement of PA (see Gandy et al., 2022, for a review). Individuals with FMS typically report low levels of PA and high levels of NA, an imbalance that may impair their ability to cope with pain (Finan et al., 2009; Hassett et al., 2008; van Middendorp et al., 2008; Zautra et al., 2005). Fostering PA may therefore be a promising strategy to improve pain outcomes and quality of life in individuals with FMS. However, current understanding of PA-based interventions for chronic pain management is limited, and few studies have examined the efficacy and mechanisms of PA-enhancing strategies in individuals with FMS (Finan & Garland, 2015). To the best of our knowledge, internet-based interventions that explicitly focus on skills for producing and maintaining PA have not been evaluated in adults with FMS; their effects on affect and pain regulation have not been examined, and evidence of underlying mechanisms of treatment effects remains unknown.

To address these gaps in the literature, the primary objective of this study was to report findings from a novel pilot intervention, Lessons in Affect Regulation to Keep Stress and Pain UndeR control (LARKSPUR). This intervention was designed to increase PA and reduce pain and fatigue in adults with chronic pain. This study reports the main analysis examining the feasibility, acceptability, and preliminary efficacy of the LARKSPUR clinical trial in adults with FMS, a chronic pain population with known physical impairments and deficits in positive emotional responses (Finan et al., 2009; Zautra et al., 2005). Dose–response analyses examine associations between intervention engagement and changes in preliminary efficacy outcomes. Our central hypothesis is that adults with FMS randomized to intervention (vs control) will show high response rate, adherence, engagement, and acceptability and report improvements in PA and pain outcomes from baseline to postintervention and postintervention to 1-month follow-up.

## Research Design and Methods

### Description of the Pilot Trial

The LARKSPUR pilot (Trial Registration: NCT04869345) has been described elsewhere (Ong et al., 2022). Briefly, persons living with FMS were enrolled after eligibility screening and informed consent. Participant inclusion criteria included: (a) access to Wi-Fi Internet connection, (b) middle-aged and older adults (age ≥50 years), (c) English literacy via self-reports of fluency and reading and writing comprehension, and (d) meeting diagnostic criteria for FMS (overall score of ≥13 the American College of Rheumatology Fibromyalgia Symptom several scale) (Wolfe et al., 2011) and/or physician confirmation of FMS. Participant exclusion criteria were kept to a minimum to maximize the generalizability of the findings and included only factors that would impede study participation: (a) moderate or severe cognitive impairment (2 or more errors on 6-item Mini-Mental State Examination; Callahan et al., 2002), (b) current behavioral treatment for chronic pain, and (c) enrollment in another chronic pain trial.

Individuals randomized to the LARKSPUR group received skills training to increase PA. The online intervention was self-guided and targeted eight PA skills over five weekly learning modules. The eight skills included (a) noticing positive events (Krause, 1988; Lewinsohn & Amenson, 1978); (b) savoring positive events (Bryant, 1989; Langston, 1994); (c) identifying personal strengths (Taylor & Lobel, 1989; Taylor et al., 1992); (d) behavioral activation to set and work toward attainable goals (Manos et al., 2010; Moskowitz et al., 2009); (e) mindfulness (Grossman et al., 2007; Kabat-Zinn, 2003); (f) positive reappraisal (Folkman, 1997; Moskowitz et al., 1996); (g) gratitude (Emmons & Crumpler, 2000; Fredrickson, 2004); and (h) acts of kindness (Brown et al., 2003; Penner et al., 2005). The control program consisted of completing daily emotion reports during the 5-week intervention period.

The control program included daily emotion reporting during the 5-week intervention period. The daily emotion reports consisted of ratings of positive and negative emotions during the past 24 hr and were similar to those used in previous studies of positive psychology interventions (Moskowitz et al., 2019). The purpose of this control condition was to match the intervention group in terms of frequency and duration of online contact, as well as attention to emotional states, without providing any specific skills or strategies to enhance PA or cope with pain.

Participants in both arms were assessed at baseline, postintervention, and follow-up. In addition, immediately before (baseline) and after the intervention (postintervention), and at 1-month follow-up, participants completed a 7-day burst of online daily positive and negative emotions. Participants were compensated for their time and incentivized to remain in the study with gift cards: $75 for baseline, postintervention, and follow-up assessments; $42 for the diary assessments ($2 each for 21 days); and $25 for postintervention feedback. The study procedures were reviewed and approved by the Institutional Review Board (IRB) at Weill Cornell Medicine.

The PA-skills intervention on which LARKSPUR is based was developed by J. Moskowitz (R34AT009685) and has been widely tested by her team in multiple studies with more than 1,000 participants (ages 16–78) coping with various life stressors—from diagnosis with a serious illness to normative daily stress (Carrico & Moskowitz, 2014; Cheung et al., 2017; Moskowitz et al., 2012, 2014, 2017). The program has been implemented in person (individually and in groups) and most recently, has been delivered online as a self-guided program for people with type 2 diabetes (Cohn et al., 2014), depression (Addington et al., 2019; Cheung et al., 2018; Moskowitz et al., 2021), HIV (Bassett et al., 2019), cancer (Cheung et al., 2017; Salsman et al., 2020), and among the general public coping with COVID-19 (Addington et al., 2022).

### Study Sample

Data were collected from 95 participants, ranging in age from 50 to 80 years old. The majority were female (94%) and identified as White (80%). Participants were recruited from July 2021 to June 2022 through referrals from NYS practicing physicians, posted flyers throughout the New York Presbyterian Healthcare System, New York City-based senior centers, community centers, and online platforms (e.g., Facebook groups). Recruitment links were also posted on clinicaltrials.gov, and emailed to potential participants via ResearchMatch, a national health volunteer research registry created by several U.S.-based academic institutions and supported by the U.S. National Institutes of Health.

### Outcome Measures

#### Feasibility and acceptability

The primary outcomes included feasibility and acceptability. Feasibility was assessed through recruitment, retention, engagement, and adherence. Engagement with the intervention was assessed for the LARKSPUR group using the number of completed daily home practices during the intervention window, the number of weeks to complete all daily home practices at least once, and intervention skill completion. In addition, adherence was assessed by examining the number of daily check-ins completed for both the LARKSPUR and control groups. Acceptability was assessed at postintervention. Participants were asked to provide feedback on perceived helpfulness, usability, and overall satisfaction with the program. Participants were also asked to rate how much they would recommend the program to someone else with chronic pain using an 11-point Likert scale, ranging from 0 = *definitely not* to 10 = *definitely yes*.

#### Preliminary efficacy

At baseline, immediately postintervention, and 1 month after intervention completion, participants were asked to complete measures of pain characteristics (i.e., pain intensity, interference, and catastrophizing) and physical functioning (i.e., activities of daily living and fatigue).

*Pain intensity* and *pain interference* were measured by the PROMIS-SI and PROMIS-PI scales. The PROMIS-SI included 3 items that asked participants to rate pain intensity over “the past 7 days” and “right now” using a 0 = *no pain* to 4 = *very severe* rating scale (Deyo et al., 2016). The PROMIS-PI included 6 items that asked participants to self-report on the consequences of pain on relevant aspects of life using a 0 = *never* to 4 = *always* rating scale (Amtmann et al., 2010).

*Pain catastrophizing* was assessed with the pain catastrophizing scale (PCS) (Sullivan et al., 1995). The PCS includes 13 items that assess three dimensions of catastrophizing: rumination (“I can stop thinking about how much it hurts”), magnification (“I worry that something serious may happen”), and helplessness (“It’s awful, and I feel that it overwhelms me”). Respondents are asked to reflect on past pain experiences and to indicate the degree to which they experienced each of the 13 thoughts and feelings when experiencing pain on a 5-point scale (0 = *not at all* to 4 = *all the time*).

*Physical functioning* was assessed using the PROMIS-PF (Rose et al., 2008) and PROMIS F-SF (Bingham et al., 2019). The PROMIS-PF includes 10 items that assess abilities and limitations concerning everyday physical activities, such as climbing the stairs, carrying groceries, and being able to sit on and get up from the toilet. Respondents are asked to report limitations and abilities to perform activities on a five-point scale, ranging from 1 = cannot/unable do to 5 = *not at all/without any difficulty*. The PROMIS F-SF is a six-item instrument that assesses the level of fatigue over a 7-day recall period. Respondents are asked to report fatigue on a scale on a five-point scale, ranging from 1 = *not at all* to 5 = *very much*.

Participants completed a 7-day burst of online daily positive and negative emotions at each pre, post, and follow-up assessment.

*Daily positive and negative affect* were assessed with the modified differential emotions scale (mDES) (Fredrickson et al., 2003). The 20-item scale asks respondents to rate how often they felt 10 positive and 10 negative emotions “during the past 24 hr” using a 5-point Likert-type scale, ranging from 0 = *never* to 4 = *most of the time*.

### Analytic Strategy

#### Sample size, power, type I error rate

Because the primary aim of this pilot study was to establish the feasibility and acceptability of the methods and procedures to be used in a larger, fully powered trial of LARKSPUR, the sample size was chosen based on feasibility indicators rather than formal power calculations for tests of between-group differences (Teresi et al., 2022; Whitehead et al., 2016). Additionally, because pilot studies are usually underpowered to achieve statistical significance at the conventional 5% threshold (Lee et al., 2014), we report 85% intervals along with effect estimates (Schoenfeld, 1980; Stallard, 2012).

Given this is a pilot study, we used Bayesian estimation rather than traditional frequentist methods because Bayesian statistics are better suited for modeling longitudinal data with relatively small sample sizes (Depaoli & van de Schoot, 2017). Bayesian estimation uses analogs to frequentist confidence intervals (CIs) and *p* values: highest posterior density intervals (HPDs) and posterior probabilities of direction (*p*_*d*_). Bayesian HPDs, like CIs, describe uncertainty around effect estimates. HPDs, however, have an intuitive interpretation (Kruschke, 2015). Specifically, the HPD summarizes the central portion of the posterior distribution that contains a certain percentage of the most probable parameter values (Kruschke & Liddell, 2018). For example, an 85% HPD of 0.25–0.50 allows one to conclude that the probability that the population effect lies between 0.25 and 0.50 is 85%. The posterior probability of direction (*p*_*d*_) for each effect or parameter was computed using the *bayestestR* software package (Makowski, Ben-Shachar, & Lüdecke, 2019). The *p*_*d*_ value is similar to frequentist *p* values (Makowski, Ben-Shachar, Chen et al., 2019). Unlike the *p* value, however, the *p*_*d*_ value directly quantifies the existence of an effect. For example, if 99% of effect values in the posterior distribution are above 0, then we would have high certainty that the data suggest that the true effect is positive. Consistent with recommendations for using less conservative significance thresholds when forming credible intervals (see Lee et al., 2014), we used a *p*_*d*_ threshold of 92.5% corresponding to a 15% significance level in the current study. Thus, measures that showed a significant pre-to-post intervention effect (*p*_*d*_ > 92.5%) and pre-to-post intervention effects that were at least small (Cohen *d* > 0.15) were considered feasible to assess the effects of the intervention in a larger trial.

#### Measurement error

Failure to account for measurement error across assessment points can result in imprecise and biased parameter estimates and intervention effects (e.g., Bollen, 1989; Loken & Gelman, 2017). In the current study, measurement error was accounted for by using a Bayesian errors-in-variables approach (Goldstein, 2008) to yield more accurate and precise effect estimates. For the PROMIS pain intensity, pain interference, physical functioning, and fatigue measures, T-scores and measurement errors are provided by HealthMeasures (2023) and were used directly to account for measurement error. For the mDES-PA, mDES-NA, and PCS, measurement error was accounted for using Williams and Hazer’s (1986) approach, which uses Cronbach’s alpha as a lower bound for reliability (reliabilities for the three measures ranged from 0.87 to 0.94). As a sensitivity check, we also conducted analyses without adjusting for measurement error; results were similar regarding within-group change and between-group differences in change.

#### Longitudinal mixed-effects models

Preliminary efficacy outcomes were assessed using intention-to-treat analyses for all participants providing data at baseline and at least one of the postintervention or follow-up assessments. Data were modeled using longitudinal mixed-effects models (Singer & Willett, 2003) and estimated within a Bayesian framework (Gelman et al., 2014) using the *brms* software package (Bürkner, 2017). Treatment group was effect coded (0.5 = LARKSPUR, −0.5 = control). We allowed for treatment differences in change by including interaction effects between time and treatment group. Planned comparisons were performed to compare (a) within-group change from baseline to postintervention, postintervention to 1-month follow-up, and baseline to 1-month follow-up, and (b) between-group differences in change from pre-to-post, post-to-follow-up, and pre-to-follow-up. We present effect sizes (Cohen’s *d*) for the within-group changes and between-group differences in change with corresponding posterior intervals. For completeness, we also present between-group differences within each assessment point as effect sizes with corresponding posterior intervals.

#### Dose–response analyses

Past research demonstrates that the frequency with which participants engage in intervention exercises significantly predicts better pain outcomes (e.g., Muller et al., 2016; Nicholas et al., 2012). To test for dose–response effects of intervention engagement (i.e., number of daily home practices completed), we fit a latent change score model (LCSM; McArdle & Nesselroade, 1994) to the data from baseline to 1-month follow-up. Figure 2 shows a path diagram of the basic LCSM for engagement and changes in outcomes. Observed variables are represented as squares; latent (unobserved) variables are represented as circles; and means are represented as triangles. The LCSM models unobserved (latent) scores at each time point as a latent variable (*f*_*t*_) defined as the sum of (a) the latent score at the previous occasion (*f*_t−1_) and (b) the latent difference $\left(\;\Delta \;f_{t-1,\;t}=f_{t}-f_{t-1}\right)$ between the two time points (McArdle & Nesselroade, 1994). We focus on the relationships between engagement and baseline to postintervention change $\left(\gamma_{E1}\right)$ and between engagement and postintervention to 1-month follow-up change $\left(\gamma_{E2}\right)$_._

**Figure 1.**
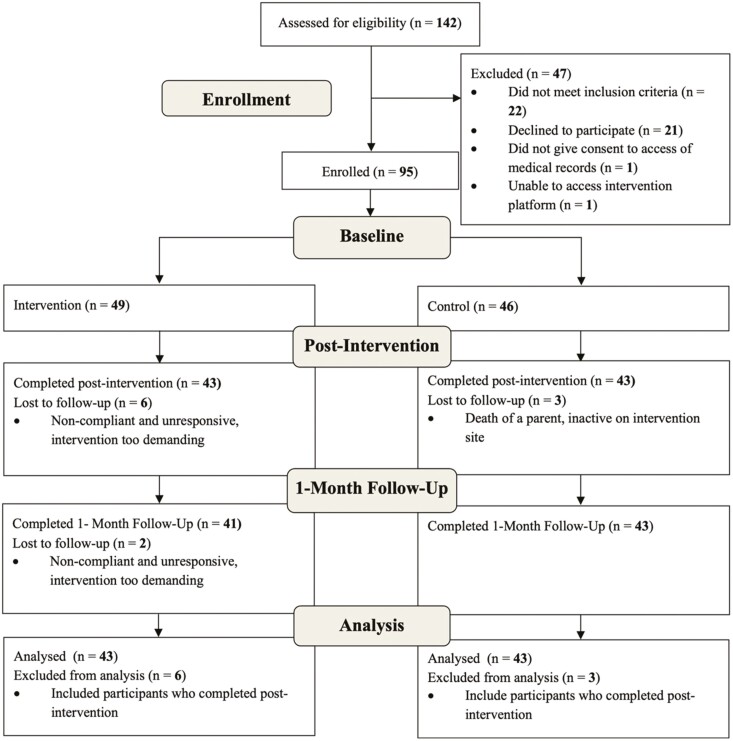
Consort diagram for LARKSPUR study. LARKSPUR = Lessons in Affect Regulation to Keep Stress and Pain UndeR control.

The LCSM provides multiple advantages for analyzing longitudinal data in randomized trials (see Könen & Karbach, 2021; McArdle & Prindle, 2008). First, change between time points can differ, resulting in nonlinear change across the study. For example, improvements may be greatest from baseline to postintervention but stable (or even reversed) from postintervention to 1-month follow-up. Second, predictors of each period of change can be included. In the current study, we evaluate engagement as a predictor of intervention effects ($\gamma_{E1}$) as well as long-term maintenance ($\gamma_{E2}$). Third, change can be modeled free of measurement error using latent variables, which is preferable to analyzing observed difference scores (e.g., Trafimow, 2015).

To accurately estimate the relationship between engagement and change in outcomes free of confounding, we used an instrumental variable approach where treatment assignment is an instrumental variable (e.g., Emsley et al., 2010; Maracy & Dunn, 2011) in the LCSM. We fit separate models for each emotional well-being and pain outcome to analyze how change in these outcomes over time differed between treatment groups. Models were estimated using the *lavaan* software package (Rosseel, 2012; version 0.6-14); 85% bias-corrected bootstrap CIs were computed for engagement-change relationships using 5,000 bootstrap replications (Preacher & Hayes, 2008; Preacher & Selig, 2012).

## Results

Of 142 individuals screened for participation, 95 were eligible and enrolled in the study. All 95 participants completed the baseline questionnaires and were randomized to LARKSPUR intervention (*n* = 49) or the control (*n* = 46; see the CONSORT diagram in Figure 1). We used an intention-to-treat analysis to evaluate secondary outcomes, which meant that participants who completed baseline and postintervention assessments were included in the analytic sample. To ensure inclusivity, we did not exclude participants who were unable to complete the 1-month follow-up assessment from our analysis. Consequently, our analytical sample (*n* = 86) included 43 control participants (out of an initial total of 49 randomized) and 43 LARSPUR participants (out of an initial total of 46 randomized), as shown in Figure 1. Demographic variables for the study sample are provided in Table 1. Because demographic measures and baseline measures were collected before randomization to treatment groups, we did not formally test for differences in demographic and baseline measures because any differences, particularly in a small pilot sample, may be the result of chance rather than due to systematic bias (Moher et al., 2010).

**Table 1.** Demographic Characteristics of Participants in the LARKSPUR (*n* = 43) and Control (*n* = 43) Groups

Variable	LARKSPUR *n* (%)	Control *n* (%)	Overall *n* (%)
Gender			
Male	1 (2)	4 (9)	5 (6)
Female	42 (98)	39 (91)	81 (94)
Age (years)			
50–59	17 (40)	22 (51)	39 (45)
60–69	19 (44)	17 (40)	36 (42)
70–79	6 (14)	4 (9)	10 (12)
_ _≥80	1 (2)	0 (0)	1 (1)
Ethnicity			
Hispanic or Latino	1 (2)	2 (5)	3 (3)
Black or African American	3 (7)	4 (9)	7 (8)
White	35 (81)	34 (79)	69 (80)
More than one race	3 (7)	1 (2)	4 (5)
Not reported	1 (2)	2 (5)	3 (3)
Marital status			
Single, never married	6 (14)	7 (16)	13 (15)
Married or domestic partnership	21 (49)	25 (58)	46 (53)
Separated	2 (5)	0 (0)	2 (2)
Divorced	10 (23)	9 (21)	19 (22)
Not reported	4 (9)	2 (5)	6 (7)
Education			
High school diploma or GED	2 (5)	1 (2)	3 (3)
Some college (no degree)	9 (21)	8 (19)	17 (20)
Associate degree	6 (14)	5 (12)	11 (13)
Bachelor’s degree	12 (28)	9 (21)	21 (24)
Postgraduate (no degree)	3 (7)	7 (16)	10 (12)
Master’s degree	9 (21)	12 (28)	21 (24)
Doctoral degree	2 (5)	1 (2)	3 (3)
Employment			
Retired	19 (44)	15 (35)	34 (40)
Unable to work	10 (23)	8 (19)	18 (21)
Employed full time	7 (16)	4 (9)	11 (13)
Self-employed	3 (7)	6 (14)	9 (10)
Employed part time	3 (7)	6 (14)	9 (10)
Unemployed seeking work	0 (0)	1 (2)	1 (1)
Unemployed not seeking work	0 (0)	2 (5)	2 (2)
Homemaker	1 (2)	1 (2)	2 (2)

*Notes*: GED = general equivalency degree; LARKSPUR = Lessons in Affect Regulation to Keep Stress and Pain UndeR control; *N* = total within column; % = percentage of total within column.

### Feasibility and Acceptability

Feasibility (recruitment, retention, adherence, and engagement) and acceptability are summarized in Table 2. Of the 120 individuals who met the inclusion criteria, 95 were enrolled, resulting in a recruitment rate of 79%. Of the 95 enrolled participants, 86 (90%) were retained through the intervention period and completed postintervention assessments. Retention rates were similar between the LARKSPUR (88%) and control groups (93%). Two participants were lost to follow-up in the LARKSPUR group. Intervention engagement (as measured by daily practice exercise completion and skill completion) was acceptable. Within the LARKSPUR group, the median time to complete all daily practice exercises at least once was 4.06 weeks (*IQR* = 0.16). Intervention skill completion was near perfect (*median* = 100%, *IQR* = 0%), and the median number of completed daily home practice exercises was 173 (*IQR* = 122), or nearly five exercises per day, indicating high intervention engagement. Overall adherence (as measured by completion of daily check-ins) was moderate; the median number of daily check-ins was 25 (21 in the LARKSPUR group, 31 in the control group) within the 35-day intervention period. At the postintervention assessment, LARKSPUR participants rated the intervention favorably on an 11-point Likert scale (0 = *definitely no* to 10 = *definitely yes*). Participants were very likely to recommend LARKSPUR to a friend (*M* = 8.89, *SD* = 1.75) and very likely to recommend LARKSPUR to someone with chronic pain (*M* = 9.14, *SD* = 1.49).

**Table 2.** Recruitment, Retention, Adherence, Acceptability, and Engagement

Variable	LARKSPUR			Control		Overall	
	*n* (%)	Median (IQR)	Mean (*SE*)	*n* (%)	Median (IQR)	*n* (%)	Median (IQR)
Recruitment rate (% enrolled)						95 (79)	
Retention (% enrolled completing postintervention)	43 (88)			43 (93)		86 (90)	
Retention (% enrolled completing postintervention and follow-up)	41 (84)			43 (93)		84 (88)	
Adherence (number of daily emotion reports)		21 (14–29)			31 (17–34)		25 (14–32)
Acceptability							
* Recommendation to friend*			8.89 (0.27)				
* Recommendation to someone with chronic pain*			9.14 (0.23)				
Engagement (weeks to complete intervention^a^)		4.06 (4.01–4.17)					
Engagement (intervention skills completed [%])		100 (100–100)					
Engagement (number of daily home practices)		173 (100–222)					

*Notes*: IQR = interquartile range; LARKSPUR = Lessons in Affect Regulation to Keep Stress and Pain UndeR control; *N* = total within column; % = percentage of total within column; *SE* = standard error. Summary statistics for number of daily emotion reports, acceptability (0 = definitely no, 10 = definitely yes), Weeks to complete intervention, intervention skills completed, and number of daily home practices are reported based on the postintervention analytic sample (*n* = 43 in LARKSPUR, *n* = 43 in control).

^a^ *n* = 3 participants did not complete all daily practice exercises and are excluded.

### Preliminary Efficacy

#### Affective well-being

Table 3 presents the model-estimated posterior means, 85% highest posterior density intervals, and effect sizes (i.e., Cohen’s *d*) of the preliminary efficacy measures at each assessment point. Planned contrasts suggested that the LARKSPUR group improved significantly from baseline to postintervention on PA, *p*_*d*_ = 100% and NA, *p*_*d* =_ 97.34%. Furthermore, improvements in PA (*p*_*d*_ = 100%) and NA (*p*_*d*_ = 99.98%) were maintained at the 1-month follow-up assessment for the intervention group. In the control group, a significant decrease in PA was found at postintervention (*p*_*d*_ = 97.34%) that was maintained at 1-month follow-up (*p*_*d*_ = 97.34%). Notably, LARKSPUR led to larger improvements in PA (*p*_*d*_ = 100%) and NA (*p*_*d*_ = 98.48%) than control, and these improvements were maintained at 1-month follow-up (PA, *p*_*d*_ = 100%; NA, *p*_*d*_ = 99.98%). Figure 3 shows the time course of PA and NA for LARKSPUR and control participants based on predicted values from the longitudinal analyses.

**Table 3.** Model-Estimated Posterior Means, 85% Highest Posterior Density Intervals and Effect Sizes (*N* = 86 in Analytic Sample)

Outcome	Baseline	Postintervention	Follow-up	Pre-to-post	Post-to-follow-up	Pre-to-follow-up
	Mean (85% HPD)	Mean (85% HPD)	Mean (85% HPD)	ES (85% HPD)	ES (85% HPD)	ES (85% HPD)
mDES-PA						
LARKSPUR	1.67 [1.42, 1.90]	1.83 [1.59, 2.06]	1.79 [1.54, 2.01]	0.15 [0.12, 0.18]	−0.04 [−0.07, −0.01]	0.11 [0.08, 0.14]
Control	1.70 [1.48, 1.93]	1.66 [1.44, 1.89]	1.64 [1.41, 1.87]	−0.04 [−0.07, −0.01]	−0.02 [−0.05, 0.01]	−0.06 [−0.09, −0.03]
L−C ES		0.15 [−0.03, 0.34]	0.13 [−0.06, 0.32]	0.19 [0.15, 0.24]	−0.02 [−0.07, 0.02]	0.17 [0.13, 0.22]
mDES-NA						
LARKSPUR	0.66 [0.43, 0.90]	0.62 [0.39, 0.86]	0.59 [0.36, 0.82]	−0.04 [−0.07, −0.01]	−0.04 [−0.069, −0.00]	−0.08 [−0.11, −0.05]
Control	0.55 [0.32, 0.78]	0.57 [0.34, 0.80]	0.58 [0.35, 0.81]	0.02 [−0.00, 0.06]	0.00 [−0.03, 0.04]	0.03 [−0.00, 0.06]
L−C ES		0.05 [−0.13, 0.25]	0.01 [−0.17, 0.19]	−0.07 [−0.11, −0.02]	−0.04 [−0.08, 0.01]	−0.11 [−0.15, −0.06]
PCS						
LARKSPUR	1.54 [1.30, 1.76]	1.18 [0.95, 1.41]	1.13 [0.90, 1.37]	−0.33 [−0.39, −0.27]	−0.04 [−0.10, 0.02]	−0.37 [−0.43, −0.30]
Control	1.25 [1.01, 1.49]	1.05 [0.80, 1.29]	0.92 [0.68, 1.16]	−0.18 [−0.24, −0.12]	−0.12 [−0.18, −0.054]	−0.30 [−0.36, −0.23]
L−C ES		0.12 [−0.09, 0.34]	0.19 [−0.02, 0.41]	−0.14 [−0.23, −0.05]	0.08 [−0.02, 0.166]	−0.07 [−0.16, 0.02]
PROMIS-SI						
LARKSPUR	63.52 [62.21, 64.82]	61.24 [59.93, 62.58]	62.00 [60.62, 63.30]	−0.50 [−0.80, −0.21]	0.16 [−0.14, 0.46]	−0.33 [−0.64, −0.04]
Control	62.66 [62.31, 63.95]	61.22 [59.90, 62.54]	61.23 [59.93, 62.56]	−0.31 [−0.60, 0.02]	0.00 [−0.30, 0.29]	−0.31 [−0.61, −0.02]
L−C ES		0.00 [−0.40, 0.42]	0.17 [−0.26, 0.57]	−0.19 [−0.60, 0.22]	0.16 [−0.26, 0.59]	−0.02 [−0.43, 0.40]
PROMIS-PI						
LARKSPUR	63.58 [62.37, 64.78]	62.11 [60.92, 63.35]	62.73 [61.46, 63.93]	−0.28 [−0.47, −0.09]	0.18 [−0.08, 0.31]	−0.16 [−0.36, 0.03]
Control	62.80 [62.61, 64.02]	62.17 [60.98, 63.41]	60.81 [59.56, 62.02]	−0.12 [−0.30, 0.07]	−0.26 [−0.45, −0.06]	−0.38 [−0.57, −0.19]
L−C ES		−0.01 [−0.34, 0.32]	0.36 [0.05, 0.71]	−0.16 [−0.43, 0.10]	0.38 [0.10, 0.65]	0.22 [−0.06, 0.48]
PROMIS-PF						
LARKSPUR	36.66 [35.38, 37.95]	38.30 [37.00, 39.60]	38.71 [37.39, 40.01]	0.29 [0.16, 0.42]	0.07 [−0.06, 0.21]	0.36 [0.23, 0.50]
Control	36.90 [35.60, 38.19]	38.22 [36.94, 39.56]	38.12 [36.84, 39.44]	0.23 [0.10, 0.36]	−0.02 [−0.14, 0.12]	0.22 [−0.08, 0.34]
L−C ES		0.01 [−0.31, 0.34]	0.10 [−0.22, 0.43]	0.06 [−0.12, 0.24]	0.09 [−0.10, 0.28]	0.15 [−0.03, 0.34]
PROMIS-F						
LARKSPUR	66.70 [64.98, 68.36]	64.08 [62.39, 65.80]	63.54 [61.84, 65.27]	−0.36 [−0.53, −0.17]	−0.07 [−0.26, 0.11]	−0.43 [−0.61, −0.24]
Control	62.81 [61.12, 64.48]	59.66 [57.98, 61.36]	61.16 [59.47, 62.86]	−0.43 [−0.60, −0.25]	0.20 [0.02, 0.38]	−0.22 [−0.40, −0.04]
L−C ES		0.60 [0.27, 0.92]	0.32 [−0.00, 0.65]	0.07 [−0.19, 0.31]	−0.28 [−0.54, −0.02]	−0.20 [−0.45, 0.05]

*Notes*: ES = effect size; 85% HPD = 85% highest posterior density intervals; L−C = LARKSPUR minus control; mDES-PA = modified differential emotions scale positive affect subscale; mDES-NA = modified differential emotions scale negative affect subscale; PCS = pain catastrophizing; PROMIS-SI = PROMIS Pain Intensity Short Form 3a; PROMIS-PI = PROMIS Pain Interference Short Form 6b; PROMIS-PF = PROMIS Physical Functioning Short Form 10a; PROMIS-F = PROMIS Fatigue Short Form 6a. PA and NA were each modeled at item level across the three seven-day bursts at each study wave using a mixed-effects model allowing for change across study waves (baseline, postintervention, and 1-month follow-up) and linear change across days within daily bursts; measurement error was accounted for by modeling inter-item variance in PA or NA item scores with random item intercepts. Effect sizes (ES) were calculated by dividing estimates on the original scale by the total model-implied marginal standard deviation (including variance among random intercepts for participants and daily within-item residual variance). PCS was modeled at the item level using a mixed-effects model; measurement error was accounted for by modeling inter-item variance in PCS item scores with random item intercepts.

**Figure 2.**
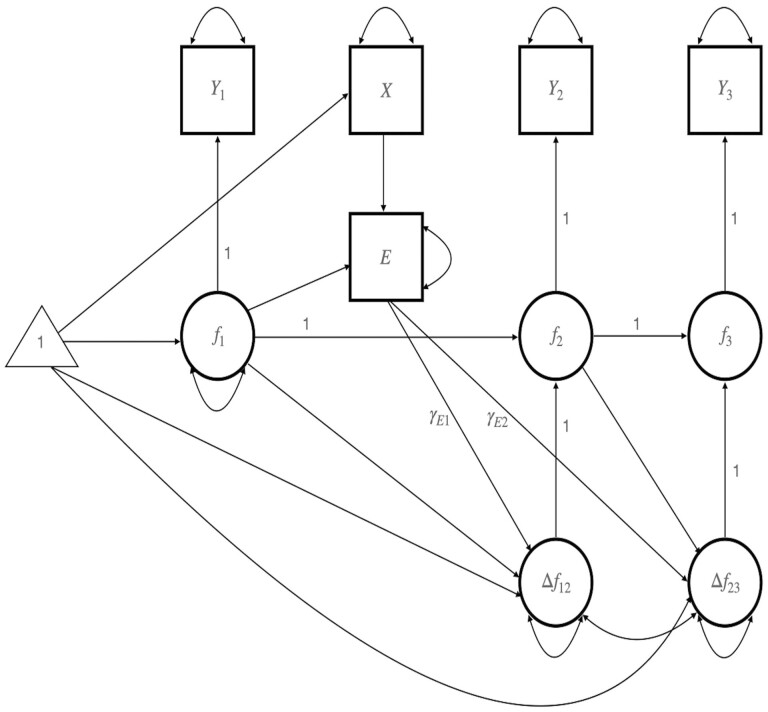
Latent change score model of intervention dose–response relationships. Engagement (E) is predicted by the treatment group (X) instrumental variable. *Y*_1_, *Y*_2_, *Y*_3_ are observed scores on an outcome at baseline, postintervention, and 1-month follow-up with corresponding latent (unobserved) variables *f*_1_, *f*_2_, *f*_*3*_ and latent change scores from baseline to postintervention ($\;\Delta \;f_{12}$) and from postintervention to 1-month follow-up ($\;\Delta \;f_{23}$). Latent variables are represented by circles. Double-headed curved arrows represent variances and covariances. Unlabeled single-headed arrows have free regression coefficients (not shown for simplicity). Paths with a fixed coefficient of 1 are labeled.

**Figure 3.**
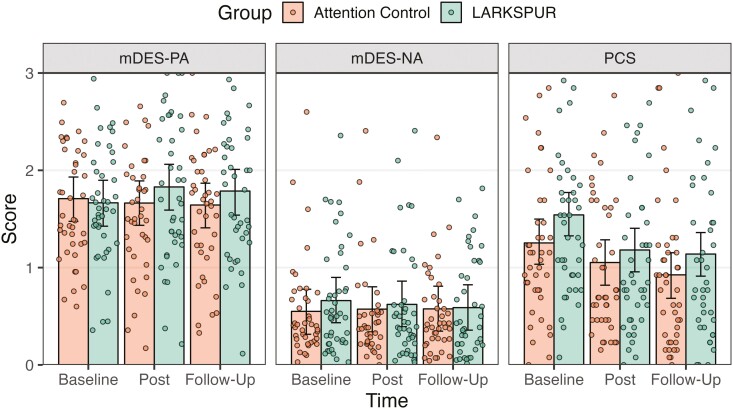
Predicted values from mixed-effects model for positive affect (mDES-PA), negative affect (mDES-NA), and pain catastrophizing (PCS) at baseline, immediately postintervention, and at 1-month follow-up.

#### Pain characteristics

Mixed-effects analyses revealed significant pre-to-post reductions in the PCS (*p*_*d*_ = 100%), PROMIS-SI (*p*_*d*_ = 99.21%), and PROMIS-PI (*p*_*d*_ = 98.22%) for the intervention group. Improvements were maintained at 1-month follow-up for PCS (*p*_*d*_ = 100%) and PROMIS-SI (*p*_*d*_ = 94.48%) but not for PROMIS-PI (*p*_*d*_ = 88.71%). Pre-to-post reductions in the control group were also found on the PCS (*p*_*d*_ = 100%) and PROMIS-SI (*p*_*d*_ = 93.84%) but not on PROMIS-PI (*p*_*d*_ = 81.87%). As shown in Figure 3, compared with control participants, LARKSPUR participants showed greater reductions in PCS from baseline to postintervention (*p*_*d*_ = 99.08%). However, improvements in pain catastrophizing were not maintained at the 1-month follow-up. No significant time-by-condition effects were found at postintervention or 1-month follow-up for pain intensity and pain interference.

#### Physical functioning

LARKSPUR participants improved significantly from baseline to postintervention on PROMIS-PF (*p*_*d*_ = 99.91%) and PROMIS-F (*p*_*d*_ = 99.75%), and these improvements were maintained at 1-month follow-up (PROMIS-PF, *p*_*d*_ = 100%; PROMIS-F, *p*_*d*_ = 99.96%). Improvements from pre-to postintervention were also observed among control participants on these measures (PROMIS-PF, *p*_*d*_ = 99.51%; PROMIS-F, *p*_*d*_ = 99.95%), which were also maintained at 1-month follow-up (PROMIS-PF, *p*_*d*_ = 99.18%; PROMIS-F, *p*_*d*_ = 96.33%). For physical functioning (PROMIS-PF) and fatigue measures (PROMIS-F), no significant time-by-condition effects were found at postintervention or 1-month follow-up.

### Intervention Dose

We examined intervention “dose” or engagement using the number of daily home practices completed. Results from LCSM indicated that engagement within the LARKSPUR intervention significantly predicted improvements in PCS, but did not predict improvements in PA or NA. For PCS, results suggested that engagement significantly predicted pre-to-post (*b* = −0.07, Bc 85% CI: [−0.14, −0.00]) and post-to-follow-up (*b* = 0.06, Bc 85% CI: [0.01, 0.12]) changes. Specifically, PCS scores decreased from baseline to postintervention (i.e., on average 5% for each standard deviation (*SD*) increase in engagement) and these changes were maintained at 1-month follow-up. For PA and NA, engagement did not predict pre-to-post (PA: *b* = 0.05, Bc 85% CI: [−0.03, 0.14]; NA: *b* = −0.03, Bc 85% CI: [−0.07, 0.00]) or post to follow-up (PA: *b* = 0.01, Bc 85% CI: [−0.05, 0.07]; NA: *b* = −0.01, Bc 85% CI: [−0.05, 0.03]) changes, although slope coefficients for both measures were in the expected direction.

## Discussion

This study reports findings from LARKSPUR, a multicomponent pilot intervention designed to improve emotional and physical functioning and reduce pain among adults with chronic pain. To our knowledge, this is the first RCT specifically targeting PA in adults living with FMS. Strengths of the study include (a) a focus on FMS, a chronic pain population with known deficits in PA; (b) a self-paced intervention delivered online; (c) use of an active, attention-matched control condition rather than a wait-list control; (d) high participant retention and engagement; (e) low loss to follow-up; (f) use of measures with strong known psychometric properties; (g) inclusion of a measurement-burst design to assess dynamic changes in PA; (h) Bayesian methods to assist in the interpretation of significance thresholds in pilot trials, and finally (i) use of LCSM to evaluate dose-response effects of intervention engagement.

### Feasibility and Acceptability

The current study provides initial, strong evidence supporting the feasibility of using an online PA-skills intervention with individuals with FMS. The study design and procedures were successful in enrolling, engaging, and retaining participants in the trial. Satisfaction with the program was high, and nearly all participants in the intervention arm reported feeling confident in recommending the program to friends or people with chronic pain. The high levels of engagement and satisfaction, combined with the lack of adverse reactions and low loss to follow-up, provide evidence of the acceptability of LARKSPUR as a relatively low-intensity online PA intervention for people with FMS.

Overall, although adherence (as measured by completion of daily check-ins) was moderate, engagement (i.e., number of daily home practices completed) was good. Feedback from participants revealed the need for assistance overcoming barriers to completing the intervention, such as difficulties practicing multiple skills within a week and difficulties completing the intervention when fatigue and pain levels were high. These comments suggest that additional attention should be given to strategies for optimizing program delivery (e.g., reducing the number of skills to assist in practice and reduce fatigue). Overall, the findings are consistent with the prior literature, which has demonstrated the value of PA-skills training in populations coping with chronic or severe health conditions (e.g., Bassett et al., 2019; Cheung et al., 2017; Cohn et al., 2014), and extend previous results in suggesting that LARKSPUR may be an acceptable form of treatment for improving quality of life and decreasing distress for people with FMS.

### Preliminary Efficacy

Results were promising with respect to the effects of LARKSPUR on preliminary efficacy outcomes. Within-person changes from baseline to postintervention indicate treatment benefits across all outcomes assessed. Moreover, improvements in PA, NA, pain catastrophizing, pain intensity, physical functioning, and fatigue were maintained at the 1-month follow-up for the intervention group. These results are particularly encouraging in that they indicate the potential of a nonpharmacologic intervention to improve symptom management in a patient population with moderate-to-severe pain. Participants in the control group also reported pre-to-post improvements in pain catastrophizing, fatigue, and physical functioning and pre-to-follow-up improvements in pain catastrophizing. Although within-group benefits were found across outcomes, between-group differences favoring the LARKSPUR group were found only on pre-to-post PA, NA, and pain catastrophizing measures. We did not assess outcomes during the intervention window to reduce participant burden, and it is possible that this pragmatic design choice resulted in reduced estimates of efficacy. Studies that use intensive longitudinal designs (e.g., daily diaries) to capture variations in pain and physical functioning (Mun et al., 2019) during the intervention period may be necessary to demonstrate the sequelae of intervention effects. Finally, beneficial effects were maintained at 1-month follow-up for PA and NA; however, LARKSPUR did not lead to sustained improvements in pain or physical functioning compared with the control group. The absence of a post-to-follow-up effect of the intervention on pain and physical functioning is consistent with research that finds interventions that exclusively focus on increasing PA may be insufficient to reinforce long-term efficacy in pain outcomes (i.e., Hausmann et al., 2018).

With respect to our aim to identify appropriate outcome measures for a full-size randomized trial (Cohen *d* > 0.15), the findings from the current pilot trial indicate that all outcome measures except for those assessing NA would be appropriate and feasible. The LARKSPUR intervention may simply not be effective in reducing NA (Cohen *d* = −0.04). This is consistent with findings that among people newly diagnosed with HIV, a PA intervention resulted in no significant reductions in NA (Moskowitz et al., 2017). It is also possible that the measure chosen to assess NA may not be sensitive enough to detect the effects of the intervention in a relatively small sample. Furthermore, although the effect sizes for measures of pain (*d* = −0.28 to −0.50) and physical functioning (*d* = 0.29 and −0.36) in this study were comparable to those reported in a recent meta-analysis of internet-delivered interventions for chronic pain (Gandy et al., 2022), the pre-to-postintervention effect size for PA was small (*d* = 0.15) in this study. One potential reason for this small effect is that the control condition, which involved emotion reporting, may have had an active ingredient that improved PA (see Moskowitz et al., 2017). Therefore, the lack of strong effects on PA in this study could be due to benefits experienced in the control condition rather than a limitation of the intervention.

### Dose–Response Relationships

We examined data on the frequency of home practice completion to gain more insight into dose–response relationships. Consistent with prior research (Muller et al., 2016; Nicholas et al., 2012), results from the current study indicated that engagement (daily home practice) led to reductions in pain (i.e., PCS), but not affective outcomes (i.e., PA or NA). It is possible that for the skills to have the greatest impact, they would need to be practiced more frequently and for a longer duration (i.e., postintervention) than they were practiced by participants in this study. Future studies that are powered to test multiple mediation pathways between PA interventions and pain-related outcomes should examine these and other potential mechanisms.

### Limitations and Future Directions

Several limitations provide direction for future research. As this was a feasibility study, the sample size was small, and the study was not designed or powered to detect meaningful changes in pain outcomes. However, the effect size and variability estimates in LARKSPUR may help to plan appropriate sample sizes for future trials. Our study sample was restricted to adults with FMS, limiting our findings’ generalizability to adults with other chronic pain conditions. Furthermore, as is common in research with FMS (Bennett et al., 2007; Davis & Zautra, 2013), most of the participants in this study were women, and a large proportion was White, educated, and married. The study also relied on self-report of FMS diagnosis due to privacy constraints in accessing medical information and the study design (i.e., patient referrals from NYS practicing physicians). Future research with more diverse samples recruited from medical settings with confirmed FMS diagnosis would be beneficial.

It would be helpful in future studies to compare LARKSPUR to a more active control condition (e.g., CBT-P) to explore the extent to which outcomes could be attributed to PA enhancement versus NA minimization (Finan & Garland, 2015). We did not monitor for pain medication use during the study, which could have potentially influenced the uptake of PA skills in LARKSPUR. Future iterations should consider asking participants to report pain medication use at each assessment point. Finally, the study design did not allow for an evaluation of the specific beneficial effects of intervention tailoring (Muller et al., 2016). Here, our findings suggest that interventions targeting PA enhancement should be a priority for improving the substantial deficits in PA that are common among individuals with FMS (Finan et al., 2009). Future research could examine this issue by comparing the potential effects of individual PA skills with each other (e.g., gratitude vs behavioral activation) and evaluating them against active control groups.

## Conclusion

The results of this 6-week pilot study support the feasibility, acceptability, and preliminary efficacy of an internet-delivered PA-skills intervention in individuals with FMS. Sustained improvements in daily emotional functioning were demonstrated, whereas effects on pain and physical functioning were more limited. The study further demonstrated the feasibility of engaging and retaining people with FMS through an online-delivered intervention. A larger, fully powered RCT is warranted to advance understanding of underlying theoretical mechanisms, effects on longer-term outcomes, and benefits across clinical and demographic subgroups.
